# “It’s Always Been a Second Class Cancer”: An Exploration of the Experiences and Journeys of Bereaved Family Carers of People with Sarcoma

**DOI:** 10.3390/cancers13112670

**Published:** 2021-05-28

**Authors:** Moira O’Connor, Greta Smith, Ashleigh Pantaleo, Darren Haywood, Rhys Weaver, Georgia Kb Halkett

**Affiliations:** 1 WA Cancer Prevention Research Unit, School of Population Health, Discipline of Psychology, Faculty of Health Sciences, Curtin University, Perth, WA 6102, Australia; greta.smith@graduate.curtin.edu.au (G.S.); ashleigh.pantaleo@curtin.edu.au (A.P.); darren.haywood@curtin.edu.au (D.H.); 2 School of Nursing, Faculty of Health Sciences, Curtin University, Perth, WA 6102, Australia; rhys.weaver@curtin.edu.au (R.W.); g.halkett@curtin.edu.au (G.K.H.)

**Keywords:** sarcoma, carers, bereavement, qualitative, family

## Abstract

**Simple Summary:**

Sarcomas are a group of rare and aggressive cancers, which develop in bones and connective tissue throughout the body. Sarcomas are different to other types of cancer. Differences include the fast progression of the cancer and late diagnoses. This leads to distress for patients and carers, which can lead to negative experiences for carers after bereavement. In this this study we aimed to explore the experiences of bereaved family carers of people diagnosed with sarcoma. We conducted interviews with sixteen bereaved carers and found that bereaved carers thought about their experiences as a journey.

**Abstract:**

Sarcomas are a group of rare and aggressive cancers, which develop in bones and connective tissue throughout the body. Sarcomas account for only 1–2% of all cancers worldwide; however, mortality rates for sarcoma are high with approximately two in four sarcoma patients dying following a diagnosis. Delays in diagnosis, poor management of symptoms, patients’ high symptom loads and high carer burden are all associated with carer distress, which may lead to complications after bereavement. The experience of having a family member referred for palliative care is also distressing for carers, with the realisation that their family member is dying. This study aimed to explore the experiences of bereaved family carers of people diagnosed with sarcoma. A qualitative descriptive design using a social constructionist framework was adopted. Interviews were conducted with sixteen participants, and thematic analysis was used to identify patterns in the data. Four overarching themes emerged: beginning the journey; moving through treatment; transitioning to palliative care; and experiencing bereavement. The narratives were coherent and potent, and people reflected on their journeys. Interventions and supports for bereaved carers could include opportunities for counselling to support reflections, supports for developing a narrative such as writing therapy, and preparation for the death of the family member.

## 1. Introduction

### 1.1. Sarcomas

Sarcomas are a group of rare and aggressive cancers, which develop in bones and connective tissue throughout the body [1]. Sarcomas account for only 1–2% of all cancers worldwide [2]; however, mortality rates for sarcomas are high with approximately two in four sarcoma patients dying following a diagnosis [3]. Approximately 90% of sarcomas are diagnosed in adults but sarcomas affect a much larger proportion of children and adolescents when compared to other cancers, accounting for approximately 20% of all paediatric malignant cancers [4]. Whilst there have been improvements in treatment outcomes in the last decade, the five year overall survival rate for sarcomas (combined adult and paediatric rates) remains low with five-year survival rate of bone and tissue sarcomas approximately 70% and 67%, respectively [3,5]

### 1.2. Delays in Diagnosis and Treatment

Symptomatology can be vague and intermittent. Many sarcomas go unnoticed, or are mistaken for other benign conditions, such as soft tissue sporting injuries [6]. This results in delays in diagnosis, which contribute to patient morbidity [7], larger tumours and increased likelihood of amputation [6]. Delayed diagnoses may also lead to greater patient psychological distress and lower treatment adherence [7,8]. This distress related to problems in diagnosis has been found to last up to a year after treatment [9]. Alongside this, many clinicians lack experience in treating sarcoma due to its rarity, resulting in poor management of the disease and a lack of appropriate referrals [8].

### 1.3. Carer Distress

High in-patient costs, and family/patient preferences, mean that family care at home is common [10]. Sarcoma patients are also often left with significant physical impairments leading to high levels of dependency on their carers [11]. Delays in diagnosis, poor management of symptoms, patients’ high symptom loads and high carer burden are all associated with carer distress, which may lead to complications in bereavement, such as post-loss depression and complicated grief [9].

The experience of having a family member referred for palliative care is also distressing for carers, with the realisation that their family member is dying [12]. Additionally, for many bereaved sarcoma carers, the death of their family member may have closely followed their diagnosis which can be emotionally painful [13]. Based on the World Health Organisation’s 2002 definition, in Australia palliative care is defined as: “… an approach that improves the quality of life of patients (adults and children) and their families who are facing problems associated with life-threatening illness. It prevents and relieves suffering through the early identification, correct assessment and treatment of pain and other problems, whether physical, psychosocial or spiritual.” [14], while, end-of-life refers to the last weeks of life. The WHO definition (2002) emphasises that palliative care can be provided while patients are receiving treatments with curative intent.

### 1.4. Aim

Despite the inherent challenges to carers of sarcoma patients, there is a lack of research exploring their experiences. Difficulties in recruiting participants, institutional disinterest and disproportionately low funding continue to hamper the study of rare cancers such as sarcoma [15]. However, due to the possibility of complications after bereavement, it is imperative that we explore bereaved family carers’ experiences so that we can develop and introduce appropriate interventions. This study aimed to explore the experiences of bereaved family carers of people diagnosed with sarcoma.

## 2. Materials and Methods

A qualitative descriptive design using a social constructionist framework was adopted as the research was exploratory and applied. This approach proposes that each person has a constructed version of reality and that there are multiple realities, all with equal value [16].

Sixteen participants aged 21–66 participated in the study with a mean age of 52.44 years (SD = 13.50). Fifteen participants resided in Western Australia, one resided in the Australian Capital Territory and a further participant resided in Queensland. Seven participants identified as male and nine as female. Participants were eligible if they had cared for someone who had died following a sarcoma diagnosis, could converse in English, and were over 16 years old. Participants’ demographic details are provided in Table 1.

Ethics were obtained from site (RGS0000000889) and academic institution (HRE2018-0246). Participants were recruited through a cancer charity organisation aimed at advancing community awareness and medical research into sarcoma. Flyers advertising the study were featured in the Foundation’s monthly newsletter and posted on social media sites. People who volunteered for the study were reviewed by the Foundation coordinator to determine eligibility. Eligible participants were then contacted via email to arrange a meeting and provided with the participant information sheet. After consent was obtained interviews were conducted. Interview duration ranged from 28 min to 1 h and 25 min. Mean duration was 1 h and 1 min, SD was 15.28 min. Eight interviews took place at the participants’ homes; two were conducted at a university campus; two were held at local cafes; and four were conducted over the phone. The majority of interviews were conducted one-on-one, one interview involved two family members and another involved three. All participants were provided with support service contacts following the interviews and advised to contact these if they were feeling distressed. Interviews were digitally audio-recorded and transcribed verbatim.

The interview guide (see Supplementary Materials) contained open-ended questions relating to participants’ experiences during caregiving and after bereavement including: “How did you feel supported whilst caring for your family member?”, and “To what extent did you feel prepared for your family member’s death?” Prompts and follow up questions were used to facilitate depth. The guide was reviewed by a consumer (a person with lived experience) prior to conducting the first interview. In the context of this research, the consumer had lived experience of bereavement after a family member had been diagnosed with sarcoma.

Thematic analysis was conducted according to Braun and Clarke’s guidelines [17]. Thematic analysis is a flexible analytical approach used to identify patterns within and across data [17]. The analysis was completed by authors GS, MO and AP. After transcription, the interviews were read several times by each author above to ensure familiarisation with the data. Codes were then established separately by each author, transcripts were then re-read, and broad themes and sub-themes were jointly developed. Coding or interpretation disparities between the authors were solved through discussion.

As per the consolidated criteria for reporting qualitative research [18], we report that quality was achieved through reflexive journaling and maintaining an audit trail to ensure transparency of interpretation. There was team discussion around the themes until consensus was reached. Saturation was deemed achieved when no new codes or themes emerged. There was ongoing discussion with the research team until consensus on themes was reached.

## 3. Results

Findings were organised into four overarching themes: (1) Beginning the journey; (2) Moving through treatment; (3) Transitioning to palliative care; (4) Experiencing bereavement. Participants have been de-identified with participant names replaced with pseudonyms.

### 3.1. Beginning the Journey

This theme included the following sub-themes: experiencing symptoms and receiving a diagnosis.

#### 3.1.1. Experiencing Symptoms

Bereaved carers described their family member’s symptoms prior to a diagnosis of sarcoma.

The most common symptom was pain:

“*… she started developing pain in her leg, just above her knee, and I took her to the doctor’s three times*” (BC10)

“*… the physio also thought it was some sort of groin strain so that he gave him exercises to do …*” (BC05)

Carers who were parents commonly attributed the pain to the active nature of their child stating: “*He was very athletic and since he was young, he always had some aches and pains somewhere or other so it wasn’t anything new.*” (BC05) Parents also attributed the pain to aged related causes. BC02 recalled: “*One of my cousins had growing pains when he was younger … I felt … that’s probably what it is*”.

#### 3.1.2. Receiving a Diagnosis

Most families initially consulted their General Practitioner (GP), with mixed experiences. BC05 stated: “*The doctor was good, he looked at him and he said he didn’t really know what it was, but it wasn’t a groin strain.*” However, difficulties were identified in diagnosing sarcoma, with some carers recounting their GP’s lack of consideration of possible alternatives aside from age related causes: “*… the doctor just said, “I think it’s growing pains” she said to Andrea, “Jump up in the air and bend your leg” and of course Andrea could do all that.*” (BC09).

These challenges required carers to advocate for their family member. BC02 recalled: “*He was in agony and I said, “Right, that’s enough… can you [GP] do an X-ray? Because obviously there’s something wrong here otherwise he wouldn’t be still going through this*”. In some circumstances doctors questioned the legitimacy of the pain symptoms. BC07 stated: “*…they came and said, “Is it possible that Lawrence was basically putting it on for some reason?*”

Participants considered the journey began with GPs; therefore, awareness should begin there: “*When you first start having pain, that’s where you go and the GPs need to think about it as a possibility. [We need] to get more GPs going to seminars on sarcoma so at least it’s in the back of their head if they see something.*” (BC02).

### 3.2. Moving through Treatment

This theme was about experiences during the active treatment phase. Subthemes included experiencing a lack of information, needing information and sacrificing wellbeing.

#### 3.2.1. Experiencing a Lack of Information

There was a consensus amongst carers that there is a lack of information regarding sarcoma and specific types of sarcoma. BC14 recounted: “*… information was like gold, trying to find it*.” The participants stated that GPs had limited information on the disease, “*… he [GP] said this is what I know about Sarcoma. And it was four pieces of A4 paper and I was like … This is not good.*” It also came as a surprise that cancer organisations had no information on sarcoma. BC14 recounted: “*… even [organisation] … had no information on sarcoma*”.

#### 3.2.2. Needing Information

Information needs varied. For example, BC12 stated: “*… getting more information was … helpful … I want to know what’s happening*.” (BC12). Those who sought information wanted honesty and truth from GPs. BC16 recalled: “*… I did say, “Don’t tell a lie to us. We just want the facts; we want correct information*”. There was also a feeling that knowledge was power. BC09 reflected: “*We were trying to find out all this information, as much as we can, arming ourselves basically.*”

A few participants asked questions, “*Definitely got to ask questions … you want to know more …*” (BC02). Carers depended on specialist health professionals for information about sarcoma, “*We had to rely heavily on the clinical team, on the lead surgeons, for getting specifics around the issues of sarcoma*” (BC14). Others went online to source information, but said: “*Google’s not always the right thing and, because it’s a rare cancer, it’s really hard to find information.*” (BC03). Family members also needed someone to talk to as the disease progressed, “*[Palliative care organisation] came in and had a meeting with us at home. We already knew that she was terminal, it was geared towards what she wanted to do*” (BC01).

A few carers spoke about knowing too much:

“*I think number one [challenge] was knowing too much because I knew the likelihood of what was going on. I’d come across people who’d had amputations as a result of Sarcoma, and I happened to know a family who had had Ewing’s Sarcoma … when I was told it was Ewing’s Sarcoma I knew exactly what we were in for.*” (BC01)

However, not everyone wanted information: “*… one of my other sisters doesn’t want to know anything … I think Lawrence didn’t really want to know either. He wanted to just live.*” (BC12). BC05 commented, “*I’m glad I didn’t [seek out information] because it would have been demoralising … we needed to … live in the moment and believe that things possibly would [be] okay.*”

As beneficial as knowing and understanding was, knowledge had an impact: “*… even when she was diagnosed and I knew how significant it was, I had in mind pictures of people I had looked after in that stage and that’s exactly what it was like …*” (BC13)

Participants felt medical practitioners wanted to control the flow of information: “*… they [Doctors] fed information to us on a needs basis, but I think that was to protect us and to protect Clive … we dealt with what was in front of us at any given moment. They didn’t go into too much detail.*” (BC05)

#### 3.2.3. Sacrificing Wellbeing

Carers prioritised the needs of their family member over their own needs during this phase. BC10 commented: “*She became our complete focus and that’s all I did … At the time, we didn’t even give ourselves a second thought at all because you just turn all of your focus onto the patient.*” Carers and families changed their routines and lives, “*We … basically completely changed our lives … work practices—around the needs of what Carole required*” (BC14).

Some carers said their family member tried to shield them from the burden of the disease, BC04 recalled: “*… he didn’t want to burden us with it. He wanted to carry it all himself and I said, “You can’t, mate. We’re here to help you*”.

It was not until the end of the journey that some carers realised the toll the Sarcoma journey had taken on them: “*When it was all over I realised just what toll it took on me physically … it physically drained me throughout the whole process because, I was working and I obviously wasn’t eating so I was losing a lot of weight, which I didn’t realise at the time.*” (BC16)

### 3.3. Transitioning to Palliative Care

Subthemes included communicating with health professionals, accepting palliative care, dying in comfort and dying in place of choice.

#### 3.3.1. Communicating with Health Professionals

For some, the transition to palliative was difficult, with carers experiencing a breakdown in communication with health professionals. BC01 recalled: “*We picked up the fact that she was terminal because of their body language and the way they responded to her and the way the conversations were held, without them actually having the conversation with us.*” Similarly, BC02 stated:

“*… they turned up later that day and just in front of him started going on about end-of-life care … I was really angry and I [took] her outside and I said, “Why are you coming out with this in front of him?” and she said, “Oh I thought you knew. The hospital’s meant to let you know that’s what we’re here for. We’re here for palliative care”. Now when you hear palliative, you think that’s sort of the final stages and we just didn’t think it was going to be that bad or that quick or anything like that. And she said, “That’s what we’re here for. They should have warned you,*”

#### 3.3.2. Accepting Palliative Care

Many carers said their family members expressed their desire to get the most out of the remainder of their life rather than trying to extend their life. BC04 recounted discussing treatment with his son: “*We might have to look at alternatives David. If this is not going to work, we need to look at some alternatives.” And he said, “Dad, if I can’t live, there’s no point living*”.

A few also carers expressed relief when their family member accessed palliative care:

“*We went for radiation and that was just agonising and then we brought him home and it even got to the point where his care was beyond our actual realms of being able to do. So we had to take him back to the hospital and luckily they were able to give him a bed at the Palliative Care side of it*”. (BC15).

#### 3.3.3. Dying in Comfort

The gruelling treatment regimens, in some cases, became too much for patients. They questioned the purpose of their treatment and expressed a desire for quality of life. BC03 recalled their family member saying: *“How long is this [treatment] for?” and he [Doctor] said, “Indefinitely.” David said, “I won’t be able to go anywhere or do anything. What is the point?”* In some cases, the decision to stop treatment came as a shock to carers: “*She made the unbelievable decision to stop treatment …” she said, “Look, I want quality, not quantity*” (BC11).

A few carers decided that quality of life was the priority:

“*She was in great pain so it wasn’t about how can we extend this, how can we do that? It was really quality of life, those last few weeks, make sure she was pain-free and just doing what we could to comfort her*.” (BC14)

A community palliative care service was credited with helping manage pain: “*It took a while to get the pain meds sorted so they [nurses] would come and help us with the pain meds or prescribe something different.*” (BC10)

The community service was considered invaluable, but was often not involved until very late in the disease, (the nurse said): “*… wish you’d contacted us earlier because we could’ve done so much more*”.

Carers articulated that, even with the support of the palliative care team, end-of-life could be difficult: “*And then towards the end, probably the last month or so, he was just in bed with a catheter, which was kind of … cruel*”. BC15

“*Because her bowel was blocked, the tubes into her stomach, to drain her stomach continually were continually getting blocked … So sometimes a nurse wouldn’t come so we would do that ourselves … and it’s not very nice. And even though they’re there—the last three days, it wasn’t [her]*”. BC13.

This had an impact on some carers’ mental health: “*I got a mental health plan from my GP… we were running on so much adrenalin just looking after him*”. BC15.

Some carers faced difficulties such as the perception of a lack of a lack of kindness:

“*There was [one nurse] on that night … [the nurse] came out twice and at one point gave him a needle with morphine in it but [they] kind of jabbed it in and Clive just screamed. He [the nurse] wasn’t gentle*.” BC05

This perception of a lack of sensitivity extended to communication. BC02 felt that a few health professionals needed to be more thoughtful when talking about end-of-life:

“*He [Doctor] said, “So you discussed end-of-life care?” And he was just matter of fact. I honestly just wanted to cry. I was just standing at the kitchen bench … he was just rattling off these questions and I just thought, you are the most heartless person I’ve ever come across in my life*.”

#### 3.3.4. Dying in Place of Choice

When it came to deciding where family members would spend their final days, preferences differed with some choosing to remain in hospital. BC03 stated:

“*He was in a lot of pain and getting onto the stretcher, into the ambulance, all the rest of it. He said, “Why do I need to go home?” I said, “Well, you don’t need to go home.” And home would have been here, he couldn’t have got home to the farm … He said, “I think I’ll just stay here.” So he did make the choice and at that point, I think he made the choice that he’d had enough and that was it, he wasn’t going to fight anymore*.” (BC03).

In other circumstances, family members chose to return home. BC11 recalled: “*… she made the choice to be at home; she didn’t want to go to hospital to die*.” BC05 also commented on what it was like to have their family member back home: “*…* *we left the door open so he could hear us talking and you could take your dinner in and sit on his bed and all sorts of things that you couldn’t do in the hospital*”.

Some family members wanted to return home but were unable to as they still needed considerable medical attention:

“*Her bowel was blocked … … she had three lots of tubes going in and out so that always needed management. The kids wanted to take her home but I said, ‘Look, she needs too much actual medical care to do that’*” (BC13).

BC09 remembered spending a last night with their sibling:

“*All his brothers and sisters came around … We all slept—we got a sleeping bag and slept in the lounge room [laughs] … It was one of the things that stood out most in my mind from that period, that we all just sort of hung out with him for the last night*.”

### 3.4. Experiencing Bereavement

This theme had subthemes of feelings of grief, gaining support and reflecting on the journey.

#### 3.4.1. Feelings of Grief

Carers felt that grief began well before the family member died: “*… I think you do a lot of pre-grieving … I think once we knew his prognosis was not very long … I think you probably grieve the whole time.*” (BC15). This carer also spoke about how they felt soon after the death of their family member, and the let down once the adrenalin had stopped: “*I know we had just been running on adrenalin for so long that when he did pass it was this kind of like—I know that for the first couple of weeks me and Mum were like, “What do we do?”*”

Grief was conceptualised as an individual experience: “*… for me, no, the grief was too intense to talk about it … I will find my place with it, very much in my own way*”. The way grief was processed within the family unit was also unique. BC08 stated: “*Marnie, Kate and Mia, they won’t talk about it. As soon as I mention anything, they just won’t—they still won’t—they don’t want to know about it*”. This was frustrating for BC08, who felt this was something that they (BC08) needed: “*That does frustrate me because I want—that’s one thing I want to do*”. BC02 commented: “*… they all act and react in different ways and I guess that’s also a thing that keeps your mind occupied is you’ve got to deal with how the other ones are dealing with it as well*.”

For others a positive aspect of caregiving and bereavement was that it brought the family together: “*… it definitely brought us all closer together as a family in a way*”.

#### 3.4.2. Gaining Support

Most support for carers during bereavement came from their families: “*… we’ve sort of done our own bereavement support as a family*.” (BC03) However, a few carers found comfort in the shared experiences of others, describing it as:

“*… some sort of solidarity with someone” … … when I met with the other parents, yes, I felt comforted because even though there was no answer, we were all in the same boat and we could just share experiences and just provide support to each other really*.” (BC10)

Sharing experiences also enabled carers to get a glimpse of what was to come in their journey through grief, and found comfort in this:

“*… there was a chap there and his wife had her cancer for five years … he said, “I know where you are and I know what you’re going through. You will get through it.*” (BC16).

BC15 also reflected on being able to share stories with people who had been through similar situations:

“*Actually … one of my good friend’s mother … she lost her brother to sarcoma; he had soft tissue sarcoma as well. So I guess like when you meet someone and you realise they’ve had a similar experience or story … you just share the weight together*.”

#### 3.4.3. Reflecting on the Journey

Carers questioned whether they did enough, and wondered about treatment decisions:

“*I sometimes really knock myself up a little bit about should I have done more research? Should I have investigated more? Should we have taken him to America or Melbourne where there’s more things? Should I have pushed that more?*” (BC03)

In some cases, carers commented that perhaps different treatment paths should have been taken:

“*The doctor was suggesting chemo but, do you … go through that now when you’re probably, possibly won’t work and this is the last months of your life … in hindsight now I think maybe we should have tried it.*” (BC02).

There was also acknowledgment that, in some cases, treatments should not have happened, BC06 stated: “*… in a perfect world, like I said to you, that operation on him shouldn’t have happened*”.

There was an element of regret as carers thought about lost opportunities with family. BC07 stated, “*It’s just a sad thing that we didn’t do this before [holidaying with family], but circumstances are different … I wish that we had done so many different things before*”.

Some carers reflected that individual impact of a loss went beyond the experience of grief. A new normal was now the reality which a few participants acknowledged they struggled to navigate:

“*It’s a bit like Stockholm Syndrome. You’re kind of “that’s been my whole life for two and a half years and you come to the end, you go home, she dies and there’s nothing … you’re left to pick up the pieces yourself really.*” (BC10)

BC15 reflected on how the journey had brought the family closer together:

“*I’ve had conversations with them that I never dreamt I would. If anything good has come out of it, it’s the close relationship I have with them*.”

This closeness served as a way to understand that it was not about getting over their grief, it was about learning to live with it:

“*We did become closer and that closeness was very useful moving forward after [family member] died and accommodating the loss which is to learn to live with it.*” (BC14).

When reflecting back on their feelings about sarcoma, there was a revelation that the social status of sarcoma affects public perception of the disease:

“*As I put it now, it’s always seen as a second class cancer. You’re not important enough to warrant this, you’re not important enough to warrant that, but no cancer is that unimportant. You can’t say because you’ve got a breast cancer that’s more important …*” (BC01)

## 4. Discussion

Bereaved carers described their experience of sarcoma as a journey, which started pre-diagnosis and moved through to treatment, transitioning to palliative care and end-of-life treatment, and bereavement where participants reflected on their journeys. All participants told their narratives in their own way and described their unique experiences. However, there were many commonalities and shared experiences. This narration of the journey reflects previous research on the value of constructing a narrative of a family member’s life [19,20]. It also echoes work that reports on the drive to make meaning of the death of a family member [19].

Pre-diagnosis was a challenging and frustrating time, especially with GPs’ lack of knowledge and awareness of signs and symptoms of sarcoma. This reflects previous work that found delays in diagnosis were common [7]. Younger patients may be unwilling to present their symptoms and their pain is often misattributed and assumed (by GPs, family members and patients) to be caused by lifestyle [13]. These delays and misdiagnoses need to be addressed, as early diagnosis is vital to reduce the need for radical surgeries [21], reduce distress [11] and to enhance chances of survival [22].

A lack of information also characterised the treatment phase of sarcoma, with carers stating GPs, nurses and cancer organisations were able to offer little in the way of guidance. Most participants wanted information and honesty, and also asked multiple questions. These findings support previous studies that reported a lack of information available at diagnosis and during treatment [13]. However, a few carers did not want to know what they described as “too much” and preferred to take it one step at a time. This mirrors findings from a qualitative study with cancer patients that found that not everyone wanted detailed information, preferring to maintain their everyday life and routines as much as possible and avoid information overload [23]. Sarcoma specialist nurses have an important role in providing information to families; however, they may not have the resources to support adequately every patient being diagnosed [24].

The carers we interviewed redirected their lives and emotions to focus on their family member, often sacrificing their own wellbeing. This could be a focus of future research as it is common for parents of children diagnosed with cancer to neglect their own health, which is not conducive to overall wellbeing and sustained ability to support and care [25,26].

Communication was key in the family member’s transition to palliative care. The need for sensitive and effective communication emerges consistently and frequently across a number of areas in oncology, despite many years of work developing and trialling communication skills training for oncology health professionals [27,28]. A recent study by O’Connor et al. [29] found that patients in hospitals were generally comfortable talking about end-of-life preferences in a goals of care discussion but only if the discussions are conducted sensitively and in a patient-centred way.

Family members differed in how they approached end-of-life, with a number of family members wanting to end active treatment in order to have symptom control, comfort and quality of life. Carers had mixed reactions to this. Many agreed that quality of life was the overarching priority in any decisions made; however, a few carers talked about their shock over choices to end active treatment. Trauma and shock were described as psychological reactions in a study of carers of patients reaching the end-of-life [30]. These authors stated that support was needed for this trauma, and for preparation for the death of the family member.

Most participants felt they started grieving before their family members died. They talked about grieving as soon as they heard about their family member’s prognosis [31]. Feelings of grief before death is a common experience for people caring for a family member of a person living with dementia. Holm et al. [32] concluded that carers of family members in palliative care settings experienced the same grief process prior to and after the death of the family member. This anticipatory grief has been the focus of much research. Findings from a qualitative study suggest that this type of grief results in traumatic distress from the exposure to a family member’s closeness to death, and the losses resulting from anticipated separation [33]. These demands can result in intense emotions. Breen et al. [34] suggest that anticipatory grief is complex and carers are faced with a dual process of preparation while simultaneously feeling burdened with their caring responsibilities. These authors state that, in their study, emotional preparedness was the most challenging. Breen et al. [34] stress the need for interventions and supports around death preparedness. Despite a shared understanding of grieving prior to the death, stories of reactions and ways of adjusting to life without their family member reflected the literature in that every bereaved carer expressed their own unique way of grieving [35]. Even close relatives of the family member differed in their reactions, which was frustrating for many who wanted to share their feelings, talk about the family member’s life, and share memories but they found other family members preferred to keep their thoughts and feelings private.

Interestingly, in telling the narrative of their journey, carers were very willing and able to reflect, question and look back on their journey, including decisions made. For all participants this journey did not end with bereavement, rather it was perceived to be ongoing. There were many questions about whether the “right” decision was made, and reflections on how things could have been different, both in relation to treatment and in relation to experiences and missed opportunities. This was a prominent theme and related to delays in diagnosis, and the lack of knowledge and awareness in primary care. This contributed to the strong belief that sarcoma was considered a second class cancer, and resulted in rumination on the question; “what if?” This appears to differentiate the bereavement experiences of carers of patients diagnosed with sarcoma from those whose family members were diagnosed with more common cancers.

These reflections add to the narratives and provide an in-depth insight into bereaved participants’ thinking when they had time to reflect. Health professionals can learn from these thoughts and build into their practice tools and strategies to explore options (and consequences) carefully with patients and families. Interventions could include opportunities for counselling to support this reflection and questioning to avoid rumination. Health Professionals dealing with loss and grief found value in reflective practice, and this may also be useful for bereaved carers [36]. Interventions that focus on storytelling and developing a coherent narrative, such as writing therapy [37] could also be usefully applied. This novel finding around reflection after bereavement adds to the body of work on carers of people with sarcoma.

## 5. Limitations

Our sample is fairly homogenous in that most participants were parents living in metropolitan areas and recruited through the same charity organisation and social media. Participants from rural or regional areas could have different experiences, particularly in relation to early detection, resource availability, and travelling large distances for treatment. Moreover, although saturation was reached, whereby no new themes or topics emerged, we acknowledge there may be other cohorts that we were unable to access. The sample size and nature of the research means we cannot generalise from our findings; however, this is not usually within the aims or scope of qualitative research. It is also plausible that people who were experiencing complications of grief, such as prolonged grief disorder, did not volunteer to participate. However, our sample size of 16 was reasonable for a qualitative study, especially as the population is small. Reaching saturation means we captured data that is illustrative of the experiences of people bereaved through sarcoma.

## 6. Directions for Future Research

Future research should include a national survey consisting of validated instruments to capture people’s experiences quantitatively and on a larger scale. Future research should also explore potential group differences between people’s experiences in regional and rural areas as opposed to metropolitan areas, as well as if the loss of a child or adult family member affects experiences. There is also a clear need for the development of robust interventions and their subsequent assessment for feasibility and effectiveness.

## 7. Conclusions

This study is the first to capture the journey of bereaved carers of family members who were diagnosed with sarcoma. The narratives from symptoms and diagnosis through treatments, palliative care and bereavement were coherent and potent. The reflections on the journey were insightful and could be usefully incorporated into practice so that patients and carers are given as much time as possible to consider options and the possible consequences of decisions. Interventions and support for bereaved carers could include opportunities for counselling to support reflections and self-questioning so that they do not result in rumination, interventions that support developing a narrative and that focus on storytelling, such as writing therapy, and interventions that aid death preparedness.

## Figures and Tables

**Table 1:** Bereaved Carer Demographics (n = 16).

Characteristic	Number
Age (Years)	-
Mean (SD; range)	52 (SD = 13.50, Min = 21, Max = 66)
Sex (Years)	
Male	7
Female	9
Duration as a carer (months)	-
Mean (SD; range)	25 (SD = 13.06; Min = 4, Max = 52)
Time since death (months)	-
Mean (SD; range)	51 (SD = 31.72, Min = 3, Max = 99)
Relationship to patient	-
Mother	6
Father	3
Husband or male partner	3
Sister	2
Brother	1
Daughter	1
Primary tumour location	-
Pelvis	8
Lower extremities	6
Spine	1
Torso	1

Note. SD = Standard Deviation. Min + Minimum. Max = Maximum.

## Data Availability

The data that support the findings of this study are available on request from the corresponding author. The data are not publicly available due to privacy or ethical restrictions.

## Supplementary Materials

The following are available online at https://www.mdpi.com/article/10.3390/cancers13112670/s1, Supplementary Material—Interview Schedule.
