# Supplementary material for: “It’s Always Been a Second Class Cancer”: An Exploration of the Experiences and Journeys of Bereaved Family Carers of People with Sarcoma
Source: Cancers (Basel). 2021 May 28;13(11):2670. doi: 10.3390/cancers13112670 (PMC8198001; doi:10.3390/cancers13112670)
Supplement: Supplementary file 1 [file cancers-13-02670-s001.zip › cancers-1172761-supplementary.pdf]

**Interview Schedule**

**To begin our interview, I'd like to start by asking about your family members and your experience of sarcoma from symptoms and through diagnosis?**

- Did you know anything about sarcoma prior to diagnosis?
- What were some of the biggest challenges during the diagnosis period?

**Can you tell me about what it was like for you transitioning into a caring role?**

- What were some of your responsibilities as a carer?
- Would you be able to take me through a typical day for you during this time?
- What were some of the biggest challenges for you as a carer?

**How did you feel you were supported whilst caring for your family member?**

- Emotionally; Practically; Information; Financially; Professionally
- What did you find was the most helpful? Why do you think this was?
- Was there anything else you would have liked more of?
- Were there any supports that you utilised that you found unhelpful?

**Did you have any contact with the sarcoma clinical nurse consultant?**

- Did you find this helpful?
- Was the nurse able to address any unmet needs you had?

**Did your family member receive palliative care?**

Yes …

- How were you approached by the palliative care team?
- What was this experience like for you?
- Were there any gaps in the palliative care service?

No …

- Did anyone approach you about palliative care?
- Would you have liked to be approached?

**To what extent did you feel prepared for your family members death?**

- Was there anything you think would have helped you to feel prepared?
- If felt prepared … ask to elaborate.

- How did those around you respond during this time? How did this make you feel?

**And what about after your family members death could you tell me about that?**

- Did anyone make contact with you in terms of support?
- Was this contact helpful?
- Was there anything more you would have liked at this time?

**And what about since your family members death, how has life changed?**

- What have been some of the biggest challenges?
- Has anything helped you with adjusting?

**What feedback/suggestions would you give to health professionals in terms of making contact or supporting people during bereavement?**

- Is there a certain format you would suggest?
- Within a certain time period?

**Is there anything else you would like to add?**
